# Comprehensive and innovative post-market surveillance system for atypical respiratory pathogens detecting qPCR panel

**DOI:** 10.1371/journal.pone.0349967

**Published:** 2026-08-19

**Authors:** Barbara Kosińska-Selbi, Justyna Kowalczyk, Jagoda Pierścińska, Jarosław Wełeszczuk, Luis Peñarrubia, Joanna Reniewicz, Vinay Suryaprakash, Rubi Diaz-Hernandez, Martí Juanola-Falgarona, Anna Blacha

**Affiliations:** 1 QIAGEN Wrocław Sp. z o.o., Wrocław, Poland; 2 STAT-Dx Life S.L. (A QIAGEN company), Barcelona, Spain; 3 QIAGEN GmbH, Hilden, Germany; 4 QIAGEN Manchester Ltd, Manchester, United Kingdom; Kliniken der Stadt Köln gGmbH, GERMANY

## Abstract

Atypical respiratory pathogens such as *Mycoplasma pneumoniae*, *Chlamydophila pneumoniae*, *Legionella pneumophila*, and *Bordetella pertussis* are significant contributors to global morbidity and mortality from respiratory infections, and their prevalence has increased in recent years. Their detection is complicated by unique biological features and genetic variability. This study presents a comprehensive post-market surveillance (PMS) framework for the QIAstat-Dx® Respiratory SARS-CoV-2 Panel, integrating bioinformatic workflows and an AI-powered literature review developed by QIAGEN to monitor assay performance over the last years. Inclusivity and cross-reactivity protocols were applied to assess the impact of genetic mutations on assay sensitivity and specificity. The analysis revealed high inclusivity and specificity of the panel, with only a single potentially critical mutation detected in the transposon-targeted gene at low frequency and expected cross-reactivity among *Bordetella* species. The AI-driven tool enhanced the surveillance process, especially for pathogens with limited sequence data. The findings support the continued reliability of the QIAstat-Dx® Respiratory SARS-CoV-2 Panel for diagnosing atypical respiratory bacterial infections and highlight the importance of ongoing molecular surveillance using advanced bioinformatics and AI technologies.

## Introduction

Atypical bacteria are known to significantly contribute to both upper and lower respiratory tract infections, including pharyngitis, acute bronchitis, whooping cough and pneumonia [1], which have a substantial impact on global public health due to their morbidity and mortality. In 2019, nearly 600 million cases of pneumonia and other lower respiratory tract infections, encompassing both typical and atypical pneumonia, resulted in approximately 2.5 million deaths worldwide. Atypical pneumonia constitutes a substantial proportion of community‑acquired pneumonia cases, with reported prevalence varying widely across regions, partly reflecting differences in diagnostic approaches [2]. The severity of these infections varies widely, ranging from mild, self-limiting illnesses to severe, rapidly progressing infections that may lead to respiratory failure [3,4].

In primary care, most respiratory infections affect the upper respiratory tract with viruses being the main causative agent. Approximately 5% of the cases are diagnosed with Community-Acquired Pneumonia (CAP) due to viral, bacterial and atypical bacterial pathogens, including *Mycoplasma pneumoniae*, *Chlamydophila pneumoniae*, *Legionella pneumophila* [5]. Additionally, *Bordetella pertussis,* the cause of whooping cough, is associated with disease that can be mild or severe, particularly in young unvaccinated infants and the elderly [5]. Proper identification and diagnosis of atypical bacteria require collaboration between clinicians and microbiology laboratories. This involves utilizing molecular methods, serology, urinary antigen tests, or specialized culture media. Early identification of these bacteria is crucial for effective treatment, as their treatment can differ from empiric therapy choices, particularly in the treatment of pneumonia in very young children [6]. However, identifying atypical pathogens using conventional methods, like Gram staining, is not possible due to their unique cell characteristics.

In recent years, the epidemiology of atypical bacterial respiratory infections has changed. Routine syndromic testing has shown an increase in the detection of *Mycoplasma pneumoniae* and *Bordetella pertussis* in the period following the COVID‑19 pandemic. Similar post‑pandemic re‑emergence of *M. pneumoniae* has been reported across multiple regions in large‑scale epidemiological studies. Real‑world surveillance data from the QIAsphere® platform indicate that bacterial respiratory infections increased from below 1% of positive cases in 2022 to more than 10% by 2024. This rise was largely driven by *M. pneumoniae*, with *B. pertussis* also showing increased circulation [7,8]. Molecular techniques such as PCR have been shown to be highly sensitive and specific for the detection of atypical bacterial pathogens and are now considered the gold standard for diagnosis [9]. Available commercial *in vitro* diagnostic (IVD) devices such as the QIAstat-Dx® Respiratory SARS-CoV-2 Panel [10] allow the parallel detection of the atypical bacteria species in addition to the most common viruses causing respiratory tract infections. Accurate detection of *Bordetella* species is essential for effective diagnosis and surveillance. Molecular assays targeting genomic elements with high copy numbers offer a promising approach to enhance sensitivity and reliability. To achieve competitive sensitivity levels in detecting *Bordetella* species, it is recommended to target insertion sequences (IS elements) or transposons, as they are typically present in multiple copies within the genome. This makes them excellent targets for highly sensitive PCR assays [11–16]. The detection of the IS481 transposon [10] by the QIAstat-Dx® device allows better sensitivity for the detection. However, there is strong evidence that *B. pertussis*, *B. bronchiseptica*, *B. parapertussis*, and *B. holmesii* have horizontally exchanged IS elements [15,17]. Notably, *B. holmesii* and some *B. bronchiseptica* strains exhibit actual cross-reactivity with *B. pertussis,* as they also include copies of the IS481 in their genomes Although *B. pertussis* is the primary cause of *Bordetella* infections and classical whooping cough, the other *Bordetella* species like *B. holmesii* and *B. bronchiseptica* can cause respiratory tract infections and are often found in co-infections with *B. pertussis* [11,15]. The QIAstat-Dx® Respiratory SARS-CoV-2 Panel (available as QIAstat-Dx® Respiratory Panel Plus in US) is a qualitative panel assay based on multiplex real-time reverse transcriptase polymerase chain reaction (RT-PCR) designed to detect viral and bacterial nucleic acids in nasopharyngeal swab (NPS) specimens) from patients with suspected respiratory infections. The PCR assays included in the panel were designed to accommodate genetic variability across all on-panel organisms by targeting highly conserved genomic regions, ensuring broad inclusivity and stable performance over time [18–21]. A previous publication [21] has described the importance of following Post-Market Surveillance (PMS) methodologies to verify that the performance of the device remains inclusive for all targets over time. PMS protocols are essential for monitoring pathogen genetic variability and ensuring compliance with IVDR requirements.

In this study, we present two bioinformatic *in silico* workflows, complemented by AI-powered literature search tools, jointly designed at QIAGEN to monitor the genetic variability of the key atypical pathogens to ensure QIAstat-Dx® Respiratory SARS-CoV-2 Panel accurate diagnostic performance over time. These workflows assess assay inclusivity by mapping curated gene and genome sequences to reference strains and analyzing PCR oligonucleotide (primer and labelled probe) binding regions for mismatches with genomic sequences. Their criticality is evaluated based on predefined criteria that consider mismatch position, frequency, and their potential impact on oligonucleotide binding conditions. In addition, the workflow evaluates assay cross-reactivity using BLAST-based homology searches to identify potential false positives corresponding to new unexpected non-target organisms. The objective of this study was to evaluate how natural genetic variability in *Mycoplasma pneumoniae, Chlamydophila pneumoniae, Legionella pneumophila,* and *Bordetella pertussis* may impact the sensitivity and specificity of the QIAstat-Dx® SARS-CoV-2 Respiratory Panel using innovative approach, which addresses specific Post-Market Surveillance (PMS) system requirements.

## Materials and methods

A comprehensive PMS tool developed and implemented to monitor PCR assay performance over time [21] was used in this study. This included the detection of potential false negative (FN) results through an Inclusivity protocol, and potential false positive (FP) results through a Cross-reactivity protocol. The Inclusivity protocol aimed to early detect the risk of missing true positive cases, while the Cross-reactivity protocol ensured specificity by preventing misidentification of non-target sequences. In addition to analyzing publicly available sequence data the PMS protocol includes literature analysis using Huma.ai platform. This AI-powered tool integrates natural language processing and full-text analysis to retrieve relevant literature with greater precision and coverage than manual searches, as demonstrated by Reniewicz et al. [22].

### Inclusivity protocol

The bioinformatic analysis of the potential FNs is determined by the following inclusivity workflow:

1. The collection of gene sequences and whole genomes for *M. pneumoniae*, *C. pneumoniae*, *L. pneumophila*, and *B. pertussis* was obtained from the non-redundant nucleotide (nr/nt) collection database at National Center for Biotechnology Information (NCBI). The search was conducted annually for the following periods: 1 April 2021–25 May 2022; 26 May 2022–31 March 2023; 1 April 2023–31 March 2024; and 1 April 2024–31 March 2025. These data periods correspond to the retrieval intervals used for internal annual PMS reporting of the QIAstat Respiratory assay, aligned with the product launch date. Sequences originating from non-human hosts were excluded from further analysis, except for *L. pneumophila*, based on its zoonotic condition and its ecological association with protozoa—particularly amoebae—that serve as natural hosts and reservoirs (reviewed in Newton et al. [23]). Human infection occurs through inhalation of contaminated water aerosols from environmental sources, representing an indirect zoonotic transmission pathway involving non-human hosts.
2. The selected sequences for all four targets were mapped to the reference genomes of *Mycoplasma pneumoniae* (CP008895.1), *Chlamydophila pneumoniae* (LN846995.1), *Legionella pneumophila* (CP021256.1), and *Bordetella pertussis* (CP017405.1) using a high-sensitivity mapping algorithm implemented in the Geneious Prime software version 2025.2 (Biomatters, Ltd., Auckland, New Zealand, Geneious Prime User Manual). This mapping included the annotated oligonucleotides of the QIAstat-Dx® Respiratory SARS-CoV-2 Panel. To facilitate mapping, sequences were divided based on their length, such as whole genome sequences and gene sequences [21].
3. The output assembly files were manually curated in each case to avoid accidental gaps in assignment and incorrectly aligned insertions or deletions. Only unique sequences were retained.
4. To detect mismatches in the oligonucleotide binding regions for all primers and labelled probe sequences of the target-assays in focus, FASTA files were extracted from the specific binding regions. These files were then processed using a custom-written Python script. The script was designed to systematically analyse nucleotide alignments in oligonucleotide binding regions in collected sequences. First, input FASTA files were parsed and filtered to remove duplicate sequences or those containing undetermined nucleotides represented by IUPAC ambiguity code characters. Each sequence was then compared to the corresponding reference sequence on a position-by-position basis to identify nucleotide mismatches within primer and probe binding sites. Detected mismatches were encoded into standardized patterns, which were subsequently grouped and quantified across the dataset. The script further enabled aggregation of mutation frequencies and generation of structured output files to support downstream interpretation within the PMS workflow.
5. Following the methodology described in Kosińska-Selbi et al. [21], the melting temperature of primer–target and probe–target sequences containing one or more mismatches was estimated using a custom script based on the melting package version 1.8.0 from the Bioconductor Software Packages version 3.16 (www.bioconductor.org). The script processed mismatch patterns by integrating sequence and positional information for forward primers, probes, and reverse primers, and generated corresponding oligonucleotide–target sequence variants. These sequences were subsequently used as input for melting temperature estimation under predefined reaction conditions. The specific concentrations of salts, oligonucleotides, and nucleotides were adjusted according to the QIAstat-Dx® Respiratory SARS-CoV-2 Panel cartridge configuration to ensure consistency with assay conditions.
6. The impact of mismatches was evaluated according to Table 1:

**Table 1:** Impact of mismatches on primers and probes.

PCR oligonucleotide type	Location of Mismatches	Acceptance criteria	Impact
**Forward or Reverse primer**	3’ end	One or more mismatches within the first three nucleotides in the 3’ -end	Critical to detection
	Anywhere in primer	Three or more mismatches	Critical to detection
		If two mismatches: assess the estimated melting temperature (*Tm*) of the oligo with mismatches and compare it with annealing temperature of the thermal profile used in the assay.	Critical to detection if *Tm* of the primer is more than 2 °C below the annealing temperature of the thermal profile used by the QIAstat-Dx® system or if the mismatch is an insertion/deletion.
**Probe**	5’ end	One or more mismatches within the first three nucleotides in the 5’ -end	Critical to detection
	Anywhere in probe	Three or more mismatches	Critical to detection
		If two mismatches: assess the estimated melting temperature (*Tm*) of oligo with mismatches and compare it with Tm of the primer binding to the same DNA strand.	Critical to detection if *Tm* of the probe is more than 2 °C below the melting temperature of the primer binding to the same DNA strand or if the mismatch is an insertion/deletion.

For the assessment of melting temperature (Tm), primers are considered acceptable when the predicted Tm is equal to or above the nominal annealing temperature (Ta) used in the PCR thermal profile. A lower Tm may negatively affect primer binding, amplification efficiency, and assay sensitivity, particularly near the Limit of Detection (LoD). Primers with a predicted Tm up to 2 °C below the nominal Ta may be accepted as a controlled tolerance, provided that no additional high-risk features are present and that the assay meets predefined performance requirements. This threshold reflects the uncertainty of Tm prediction and should not be considered an automatic rejection criterion. However, differences greater than 2 °C may be critical and require validation under laboratory conditions to ensure assay performance [24].

7. The frequency of observed mismatches was calculated as the percentage of sequences that contained a mismatch relative to the total number of collected sequences covering the target region. Variations were considered high risk when located at the 3′ end of primers or the 5′ end of probes [18]. Threshold of three total mismatches or a single mismatch in first three bases was applied as a conservative criterion to identify potentially critical patterns.

### Cross-reactivity protocol

The detection of potential FPs was conducted by evaluating the sequence homology using the Basic Local Alignment Search Tool (BLAST) against the NCBI database (https://blast.ncbi.nlm.nih.gov/Blast.cgi), optimized for somewhat similar sequences (blastn):

1) The primer or probe sequence was used as the query in the blastn algorithm.
2) The search was restricted to the following periods: 1 April 2021–25 May 2022; 26 May 2022–31 March 2023; 1 April 2023–31 March 2024; and 1 April 2024–31 March 2025“. Additionally, the search excluded target organisms (*Mycoplasma pneumoniae*: taxid 2104; *Chlamydophila pneumoniae*: taxid 83558 and 51291; *Legionella pneumophila*: taxid 446; and *Bordetella pertussis*: taxid 520), as well as vectors, uncultured and environmental samples sequences, and synthetic sequences.
3) Algorithm parameters were set to a maximum of 5,000 hits using the default scoring parameters.
4) Hits with less than 70% total homology were filtered out, where total homology refers to the combination of the Query Cover and % Identity parameters of the BLAST results.
5) Potential false positives were identified by detecting unique sequences matching the forward and reverse primers, and the labelled probe from the same PCR assay.
6) The following factors were considered in the assessment:
  - The likelihood of the organism’s DNA being present in human samples.
  - Potential amplicon size (<= 500 bp may indicate a potential false positive).
  - Occurrence of critical mismatches in the assay’s oligonucleotide binding sites. Sequences with critical mismatches (as defined in Table 1) were not considered potential FPs.

### Literature review using Artificial Intelligence (AI) tool

Since 2024, an additional literature review has been conducted for pathogens with fewer than 100 newly added sequences covering the assay-specific target regions during a defined surveillance period, to ensure robust evaluation QIAstat-Dx® performance. In the current study, such limited sequence availability was observed for *C. pneumoniae* and *L. pneumophila*.

The Huma.ai Platform, a literature search framework jointly developed with Huma.ai, was employed for literature collection [22]. This advanced tool utilizes a multi-algorithmic framework that integrates sophisticated caching techniques with a natural language processing (NLP) system known as the Utterance Processor (UP). UP interprets user queries and dynamically constructs a pipeline of operations to retrieve and analyze relevant scientific literature. Unlike traditional keyword-based searches, the Huma.ai Platform performs full-text analysis and identifies specific regions of interest (ROIs) within articles, significantly improving the relevance and precision of the results.

The decision to use the Huma.ai Platform was based on its demonstrated superiority over manual search methods. The platform consistently outperformed manual approaches in both the number of relevant articles identified and the consistency of yearly precision rates. It also significantly reduced the time and effort required for literature screening and analysis [22].

Databases such as PubMed Central (PMC), PubMed, Embase, and ScienceDirect were searched to retrieve information relevant to product performance, including records on genetic variability in the gene target region and descriptions of newly identified strainsor genotypes of the pathogens. The resulting articles were compared across databases, and duplicate entries across databases were excluded from further assessment. ROIs were defined as sections of the publications that included descriptions of genetic variations within the assay’s target regions. These ROIs were extracted from the full text to capture information relevant for evaluating potential impact on assay performance. Sequences obtained from the selected literature were incorporated into the dataset used for the PMS analyses.

## Results

### Inclusivity analysis

A bioinformatic analysis of oligonucleotide binding site integrity was conducted across four reporting periods. The results showed no major issues or significant concerns. Only a few genomic patterns involving single nucleotide changes were detected, and these occurred at non-critical positions that are not expected to affect PCR performance (see Table 2).

**Table 2:** Summary of potential FN results across reporting periods for atypical respiratory pathogens.

Analyzed period	Target organism	No. of sequences that cover the target region	No. of sequences with observed mutations	No. of mutations found in sequences	Findings
1 April 2021–25 May 2022	*M. pneumoniae*	0	0	0	N/A
	*C. pneumoniae*	0	0	0	
	*L. pneumophila*	0	0	0	
	*B. pertussis*	136	6	5	One non-critical 1 bp change in the binding site of the forward primer with 0.74% prevalence, Two non-critical 1 bp patterns in the binding site of the labelled probe with prevalence of 0.74% and 1.47%, respectively. Two 1 bp patterns in the binding site of the reverse primer, both with prevalence of 0.74%. **One of them was considered critical based on the described criteria (Table 1)**.
26 May 2022–31 March 2023	*M. pneumoniae*	0	0	0	N/A
	*C. pneumoniae*	0	0	0	
	*L. pneumophila*	0	0	0	
	*B. pertussis*	0	0	0	
1 April 2023–31 March 2024	*M. pneumoniae*	3,201	2	1	One non-critical 1 bp pattern in the binding site of the forward primer with 0.06% prevalence.
	*C. pneumoniae*	0	0	0	N/A
	*L. pneumophila*	128	0	0	
	*B. pertussis*	113	0	0	
1 April 2024–31 March 2025	*M. pneumoniae*	156	26	1	One non-critical 1 bp pattern in the binding site of the forward primer with 16.67% prevalence. N/A
	*C. pneumoniae*	2	0	0	
	*L. pneumophila*	70	0	0	
	*B. pertussis*	219	23	3	Two non-critical 1 bp patterns in the binding site of the labelled probe with prevalence of 10.05% and 0.46%, One non-critical 1 bp pattern in the binding site of the reverse primer with 0.46% prevalence.

**Note:** N/A, not applicable.

One exception was observed during the 2021–2022 period: a critical single nucleotide mismatch was found in the binding region of the reverse primer of the *B. pertussis* PCR assay. This mismatch, associated with the MJBH01000027.1 sequence (https://www.ebi.ac.uk/ena/browser/view/PRJNA224116), had a frequency of 0.74%. However, the high copy number of IS481in the *Bordetella* genome likely mitigates the potential impact of this pattern. In addition, this mutation has not been observed in subsequent years and appears to be an isolated event that has not propagated over time. Between April 2021 and March 2025, the analysis of *M. pneumoniae*, *C. pneumoniae*, *L. pneumophila*, and *B. pertussis* confirmed the robust inclusivity of the QIAstat-Dx® Respiratory SARS-CoV-2 Panel. These findings support the continued reliability of the panel, with no significant impact on performance. A summary is provided in Table 2, with full details available in S1 Data.

Zero values in 3^rd^ column of Table 2 mean that, for the given organism and surveillance period, no publicly available sequences covering the assay target region were found using the predefined search and inclusion criteria. In such instances zero values in subsequent columns not mean that genetic variability was absent, but rather that suitable public sequence data were not available for that period. To reduce the potential bias caused by limited sequence availability, the sequence-based analysis was complemented with an AI-assisted literature review for targets with low sequence coverage. In addition, the surveillance analysis is repeated in consecutive PMS reporting periods to monitor newly available data over time.

### Cross-reactivity analysis

The BLAST-based analyses conducted across four surveillance periods (April 2021–March 2025) to evaluate potential unexpected cross-reactivity of *M. pneumoniae*, *C. pneumoniae*, and *L. pneumophila* PCR assays included in the QIAstat-Dx® Respiratory SARS-CoV-2 Panel revealed no genetic changes producing potential false-positive results (Table 3). Although multiple sequences were initially identified during the homology analysis (S2 Data), the majority either exceeded the allowed amplicon size (500 bp) or belonged to viruses not associated with human infection. On the other hand, for *Bordetella pertussis*, no unexpected sequences were detected, except for two *B. holmesii* genomes identified in the 2024–2025 period – a known cross-reactive species.

**Table 3:** Summary of potential false positive results across reporting periods for atypical respiratory pathogens.

Analyzed period	Target organism	No. of sequences returned by BLAST	No. of Potential false positive results	Observation
1 April 2021–25 May 2022	*M. pneumoniae*	9	0	N/A
	*C. pneumoniae*	8	0	
	*L. pneumophila*	9	0	
	*B. pertussis*	3	0	
26 May 2022–31 March 2023	*M. pneumoniae*	17	0	N/A
	*C. pneumoniae*	39	0	
	*L. pneumophila*	171	0	
	*B. pertussis*	1	0	
1 April 2023–31 March 2024	*M. pneumoniae*	0	0	N/A
	*C. pneumoniae*	22	0	
	*L. pneumoniae*	7	0	
	*B. pertussis*	1	0	
1 April 2024–31 March 2025	*M. pneumoniae*	0	0	N/A
	*C. pneumoniae*	12	0	
	*L. pneumophila*	1	0	
	*B. pertussis*	2	1	Cross-reactivity with sequence belonging of *B. holmesi.*

**Note:** N/A, not applicable.

### Enhancing inclusivity analysis through AI-driven literature review

To complement the sequence analysis for *C. pneumoniae* and *L. pneumophila*, a literature search was conducted using the Huma.ai Platform. Articles identified by the AI tool are provided in S3 Data.

For *L. pneumophila*, nine publications were retrieved: one from Embase, four from PMC, and four from ScienceDirect. Of these, six were considered potentially relevant based on the ROI extracted by the Huma.ai Platform, resulting in a precision rate of 66.67%. Full-text analysis revealed that all contained information about mutations in the assay target gene. However, none of the publications reported genomic variability that could impact QIAstat-Dx® performance.

For *C. pneumoniae*, 35 publications were retrieved: 33 from PMC and two from the ScienceDirect database. Of these, 22 were considered potentially relevant based on ROI, yielding a precision rate of 62.86%. Full-text analysis revealed that 18 publications contained information on genetic variability in the assay target gene. No publication reported a new *C. pneumoniae* strain or genotype, and none contained information that could impact QIAstat-Dx® assay performance.

In summary, the Huma.ai Platform successfully collected critical information to complement the sequence inquest inclusivity analysis – information that would not have been captured if the analysis had relied solely on database sequences. No additional findings indicating potential safety or performance concerns for the QIAstat-Dx® assay due to genetic variability in the target pathogens were identified, confirming its reliable performance.

## Discussion

This study assessed whether natural genetic variability in four atypical respiratory bacterial targets—*M. pneumoniae*, *C. pneumoniae*, *L. pneumophila*, and *B. pertussis*—could compromise the analytical performance of the QIAstat‑Dx® Respiratory SARS‑CoV‑2 Panel over multiple surveillance periods. Overall, the PMS framework provided convergent evidence of stable assay performance: (i) no recurring or high‑frequency critical mismatches were identified in assay oligonucleotide binding regions, and (ii) no unexpected off‑target organisms were detected that would indicate emerging cross‑reactivity risks beyond known, biologically plausible signals within *Bordetella*. These findings support the use of structured, continuous PMS to maintain confidence in molecular diagnostics as pathogen genomes evolve and as clinical need fluctuates over time.

### Clinical utility and rationale for continuous PMS

As discussed in the introduction, the post-pandemic period has been associated with increased circulation of atypical bacterial respiratory pathogens, particularly *Mycoplasma pneumoniae* [8]. This broader epidemiological trend is also reflected in real-world diagnostic data generated by routine syndromic testing. Analysis of the internally monitored QIAstat-Dx® Respiratory SARS-CoV-2 Panel cartridge results collected via the QIAsphere® platform indicates a substantial increase in bacterial detections over time, rising from below 1% of positive cases in 2022 to over 10% by 2024 [7]. Together, these observations underline the growing clinical relevance of accurate molecular identification of atypical bacterial infections and further support the importance of continuous post-market surveillance to ensure sustained diagnostic performance as pathogen circulation patterns change.

Reliable detection of atypical bacterial respiratory pathogens has direct clinical and public health implications because these infections can present with non-specific symptoms and require targeted diagnostic confirmation to support timely management and appropriate antimicrobial selection. In the post-pandemic period, multiple respiratory pathogens have shown changes in circulation patterns, including re-emergence of *M. pneumoniae* and increased *B. pertussis* activity in several settings, reinforcing the need to ensure that widely used molecular diagnostics remain fit-for-purpose as pathogen epidemiology shifts [25,26]. In this context, a robust post-market surveillance system provides an evidence-based mechanism to detect early signals of potential assay vulnerability (e.g., emerging mismatches in oligonucleotide binding regions or novel cross-reactive sequences), thereby reducing the risk of undetected loss of sensitivity or specificity during periods of heightened clinical need.

### Inclusivity analysis

Overall, the inclusivity assessment identified only limited binding-region variability and no evidence of a sustained, high-risk drift that would be expected to compromise assay detection. The most relevant signal was observed for *Bordetella pertussis* in the earliest surveillance window, where a single, low-frequency (<1%) mismatch pattern classified as potentially critical occurred in a reverse-primer binding region (MJBH01000027.1). Given its single occurrence, it remains unclear whether this represents a true biological variant or a sequencing artifact; moreover, even if genuine, the multi-copy nature of the IS481 target provides a plausible buffering context that may reduce the impact of isolated primer-site events on overall detection. Low-frequency variation in *B. pertussis* has been reported previously and contributes to genomic diversity that can be relevant for diagnostic performance [27].

For *Mycoplasma pneumoniae*, variability within the evaluated oligonucleotide binding region remained non-critical according to the applied criteria, including in the most recent dataset where a higher proportion of sequences carried a non-critical pattern. Such observations are compatible with ongoing genetic variability and align with prior reports discussing the diagnostic impact of point mutations in *M. pneumoniae* [28]. At the same time, fluctuations in publicly available sequence volumes and submission practices can influence apparent frequency changes; therefore, these observations reinforce the value of continuous monitoring rather than indicating a confirmed shift in assay vulnerability.

In the most recent window, *B. pertussis* also showed renewed non-critical variation in probe and primer binding regions. Continued monitoring is warranted, consistent with recent reports describing macrolide resistance and primer mismatches in circulating *B. pertussis* strains [29]. In contrast, no inclusivity-relevant signals were identified for Chlamydophila pneumoniae or *Legionella pneumophila* in the available datasets, and this absence of findings was corroborated through the complementary literature analysis using the Huma.ai platform. Collectively, these results emphasize that longitudinal PMS focused on oligonucleotide binding integrity remains essential to detect early signals should mutations emerge in critical positions and expand over time.

### Literature review and AI integration

For pathogens with insufficient sequence data during the last surveillance period inclusivity analysis was supplemented by literature searches using the Huma.ai platform – jointly developed by QIAGEN and Huma.ai. The tool’s performance was previously validated by Reniewicz et al. [22], demonstrating superior results compared to manual searches in terms of article retrieval, precision, and time efficiency. In this study, platform access was expanded to include two additional databases: Embase and ScienceDirect. Precision rates for *C. pneumoniae* and *L. pneumophila* (62.86% and 66.67%, respectively) were comparable to those reported by Reniewicz et al. [22] (65.14% ± 2.07% for technical assessment of the NeuMoDx HIV-1 Assay). No safety or performance concerns for QIAstat-Dx® assays were identified, corroborating sequence-based findings. Although recent studies have explored AI applications in post-market surveillance for medical devices [30,31], the current study and Reniewicz et al. [22] remain, to our knowledge, the only reports demonstrating successful NLP-based literature review for *in vitro* diagnostics. Consequently, direct comparisons between Huma.ai and other tools are challenging due to the lack of equivalent studies.

### Cross-reactivity analysis

Cross-reactivity was evaluated across four surveillance periods using BLAST-based analysis for each target organism. During the first three intervals (April 2021–March 2024), no potential false positives, defined as unexpected off‑target organisms, were detected, supporting high analytical specificity. In the final surveillance period (April 2024–March 2025), two *B. holmesii* sequences were identified for the B*. pertussis* target. These sequences contained the IS481 insertion sequence, which is shared among multiple *Bordetella* species, and this finding is consistent with the QIAstat‑Dx® specifications acknowledging potential IS481‑related cross‑reactivity (QIAstat‑Dx; QIAGEN® GmbH, Hilden, Germany). Because IS481 is not species‑specific, detection signals compatible with *B. pertussis* may also reflect other IS481‑carrying *Bordetella* species, such as *B. holmesii*, as previously reported in nasopharyngeal specimens from patients investigated for pertussis [12]. Large‑scale evaluations of IS481‑based PCR diagnostics have shown that, despite this lack of species‑level discrimination, overall clinical reliability remains high. In a U.S. multicenter study of nearly 4,000 IS481‑positive specimens, *B. holmesii* accounted for only approximately 1–2% of cases, with overall agreement between commercial assays and reference testing exceeding 85% [32]. While *B. pertussis* remains the primary etiologic agent of pertussis, occasional misclassification between *B. pertussis* and *B. holmesii* may have clinical and public health implications, including differences in expected antimicrobial susceptibility and downstream case‑management measures such as post‑exposure prophylaxis and infection‑control actions. Therefore, results for *Bordetella* targets should be interpreted in the context of known IS‑element sharing across species and, where appropriate, complemented by methods enabling species‑level discrimination. Overall, these findings confirm the assay’s specificity while highlighting the importance of continuous post‑market surveillance to monitor known and emerging cross‑reactivity patterns.

## Conclusion

This study demonstrates the robustness and reliability of the QIAstat-Dx® Respiratory SARS-CoV-2 Panel in detecting atypical respiratory bacteria – *M. pneumoniae*, *C. pneumoniae*, *L. pneumophila*, and *B. pertussis* – across four years of post-market surveillance and monitoring. The bioinformatic inclusivity analysis revealed no critical mutations for *M. pneumoniae*, *C. pneumoniae*, or *L. pneumophila*, and only a single critical mutation for *B. pertussis* occurring at low frequency in sequence databases and isolated over time, suggesting minimal risk of false-negative results. Cross-reactivity assessments confirmed robust assay specificity, showing the expected cross-reactivity among *Bordetella* species. Importantly, these findings indicate that immediate laboratory verification is not required, as the observed mutations and cross-reactivity patterns do not compromise assay performance. However, continued molecular surveillance remains essential to promptly identify any future variants that may necessitate confirmatory testing in the laboratory. Integration of AI-powered literature mining tools, such as the Huma.ai Platform, further enhanced the surveillance framework by compensating for limited sequence availability, particularly for *C. pneumoniae* and *L. pneumophila*. This approach enabled comprehensive coverage and timely identification of emerging genetic variations. Moreover, the Huma.ai Platform identified additional relevant findings that were not captured through the false negative sequence analysis alone.

Overall, the findings support the continued use of the QIAstat-Dx® Respiratory SARS-CoV-2 Panel as a reliable diagnostic tool for atypical bacterial respiratory infections, and to discriminate against them from the usual viruses causing similar symptoms. The study also underscores the importance of continuous molecular surveillance with the application of advanced bioinformatics and AI tools to proactively monitor assay performance in the face of evolving pathogen genomes. This framework offers a scalable strategy to enhance Post-Market Surveillance (PMS) processes in molecular diagnostics, enabling continuous monitoring and swift adaptation to evolution of pathogen genomes. These measures are critical to ensure accurate diagnostics and safeguard Public Health.

## Supporting information

S1 Data Full inclusivity analysis results. (XLSX)

S2 Data Full cross-reactivity analysis results. (XLSX)

S3 Data Publications identified by the Huma.ai Platform. (XLSX)
